# Vapor of Iodine in the Treatment of Ophthalmia

**Published:** 1855-12

**Authors:** Calvin G. Page


*Vapor of Iodine in the Treatment of Ophthalmia.* Reported at a meeting of the Society for Medical Observation, October 15, 1855, by Calvin G. Page, M D.

Case 1.—John Williams. Married. Coal-heaver, aged 35—a dispensary patient. First applied to me in March, 1855, for treatment of his eyes. He had partial capsular cataract of one eye, with absence of the crystalline lens, caused by a wound with a pointed arrow when he was a boy. The lids of both eyes were swollen and everted, and covered with exuberant granulations. There was great intolerance of light, pain and chemosis, with sclerotic and conjunctival injection, and constant lachrymation with some discharge of pus. I commenced using freely the sulphate of copper with cooling applications, and in a fortnight he was able to work a little, which he had not done for four months previously. Dr. H. W. Williams saw him with me about this time. He has been under my care since that time, but the improvement would not progress beyond a certain point, though all means known to me to be used in such cases were applied. His constant exposure to coal dust probably prevented the usual action of remedies. I lost sight of him during the month of August and the early part of September.

About the middle of September he again applied to me, when the condition of the eyes was as follows: The lids of both were somewhat swollen, the inner surfaces were covered with granulations; there was some injection of both conjunctiva and sclerotica, intolerance of light, dimness of vision, and lachrymation with a small amount of pus. All other means having failed, I availed myself of the fact that iodine when dissolved in chloroform, evaporates without leaving the stain of iodine, and I determined to apply this vapor to his eyes. I commenced using it on the 20th of September, and continued it daily until the 29th. After two applications the injection about the eyeball disappeared, leaving it in a perfectly normal condition. At the end of eight days there was a spot near the inner canthus, on the upper lid of each eye, entirely free from granulations. He has been seen seven times since the 28th of September, and the vapor has been applied. The granulations have nearly all disappeared from the upper lids, except at points near the outer angle. There is no intolerance of light, and the dimness of vision has disappeared.

Case 2.—Annie Fowler, aged 11 years, No. 11 Friend street, a dispensary patient, was sent by a benevolent lady to Dr. Reynolds, Sen., who sent her to the Eye and Ear Infirmary, where she was somewhat benefitted. She has scrofulous tarsal ophthalm.ia Her mother, seeing the benefit to Williams, (the patient first mentioned,) requested me to take charge of her. I have applied the vaper eight times. One eye is nearly well, the other very much improved. Both ihese patients are still under treatment.

The advantages of this method of applying iodine seem to me to be that the effect of the agent is obtained more rapidly and without the usual discoloration. The sensations to the patient are not disagreeable; the effect of smarting, &c., passes away in less than a minute. In applying it to the eyes, the lids should be closed. The vapor seems to penetrate through them. It appears to be applicable wherever iodine is called for, as in scrofulous glands, hydrops articuli, &c. The atmosphere should be excluded from the surface during the application of the vapor. —*Boston Medical and Surgical Journal.*

